# Our Journal

**Published:** 1853-09

**Authors:** 


### OUR JOURNAL.

It is peculiarly gratifying to be able to assure our numerous readers, of the triumphant success of our Journal. If a doubt, as to its future success were entertained by any one, it is now entirely dispelled. Almost every mail brings orders for it, from our kind professional brethren, to whom we are profoundly grateful and to whom we shall endeavor to render the *quid pro quo*, by making it more and more useful, interesting and instructive. A few, (we would not particularize) whom at this moment we have in our mind's eye, have expressed themselves so warmly and ardently in favor of the Journal, and the success of the Medical College, that we trust we shall never prove so recreant to the principle of gratitude or friendship, as ever to forget them. But why not thus interest themselves, and why not speak the words of encouragement? The school is a State Institution, the only one within its limits; it belongs to the people, and the people are sustaining it, that the medical profession therein, may occupy as proud a position for usefulness and efficiency as in any other State in the country. We, as the Faculty, are but the humble instruments in the hands of the people to develope its benefits and diffuse them abroad throughout the State. The Journal is the organ and exponent of the School, is a part of itself and necessary to its existence and success. It is intended to subserve no individual or selfish interest, but to promote the good of the Institution, and thereby to exercise a reflex benefit upon Medical Science and the profession at large. No one therefore who loves his profession and is the friend of medical science can feel indifferent to the success of such an enterprise.

To our fellow citizens of the City who have so generously responded to our efforts, we scarcely know what to say. Within a few days after the appearance of the first number of the Journal, more than one hundred *bonafide* subscribers were added to the list. Of course these were composed of all classes of our citizens, which act of noble generosity speaks for itself, and clearly shows their determination to stand by the Faculty in their efforts to make the Institution what the best interests of the profession require of it, and all that its many devoted friends desire to see it.

We repeat that we know not what to say, and yet we could not say too much in return for the liberality and generosity ever manifested by our good citizens toward the College.

While it is most gratifying to our feelings, it serves to awaken within us an unquailing determination, and an unconquerable resolution, that, come what may, and from whatever source, we will press forward in the discharge of duty, as long as we receive such emphatic approvals of the justness and utility of our cause. Under such influences we have endeavored to make this, the second Number, more interesting than the first, by introducing some new features into its pages; and, we assure the reader that it will be our aim in the future, to make each number an improvement upon the last.

